# Effects of sustained Trendelenburg position on the spectral signatures of the EEG: implications for the consistency of the level of anesthesia, an observational study

**DOI:** 10.1007/s10877-025-01403-x

**Published:** 2025-12-22

**Authors:** Iñigo Rubio-Baines, Antonio Martinez-Simon, Miguel Valencia, Alfredo Panadero, Elena Cacho-Asenjo, Oscar Manzanilla, Manuel Alegre, Jorge M. Nuñez-Cordoba, Cristina Honorato-Cia

**Affiliations:** 1 Department of Anesthesia and Critical Care, Clínica Universidad de Navarra, 36 Pio XII Av., Pamplona, 31008 Spain; 2 Physiological Monitoring and Control Lab, Universidad de Navarra, CIMA, 36 Pio XII Av, Pamplona, 31008 Spain; 3 Clinical Neurophysiology Section, Clínica Universidad de Navarra, 36 Pio XII Av., Pamplona, 31008 Spain; 4 Central Clinical Trials Unit, Clínica Universidad de Navarra, 36 Pio XII Av., Pamplona, 31008 Spain; 5 IdiSNA, Navarra Institute for Health Research, Pamplona, 31008 Spain

**Keywords:** Electroencephalogram, Bispectral index, Level of anesthesia monitors, Trendelenburg, Frequency bands, SEF95

## Abstract

Measurements from level of anesthesia monitors can be influenced by various factors. This study aimed to compare the evolution of BIS Vista™ monitor and electroencephalogram (EEG) parameters over time during steep Trendelenburg positioning. In this prospective observational study, data were collected from patients undergoing robot-assisted radical prostatectomy. BIS Vista™ monitor parameters (BIS Index, SEF95, EEG-BIS) and a 4-electrode EEG setup (EEG-BrVis) were recorded at baseline, 30 min after Trendelenburg positioning, and every 60 min until procedure completion. Parameter changes from baseline and trends over time were analyzed. Eighteen patients were analyzed. The mean surgical duration was 269 ± 69 min, with Trendelenburg angles ranging from 32º to 41º. No significant changes were observed in BIS Index or SEF95 in trend over the time. Mean changes in BIS Index were 0.017 ± 0.01 (*p* = 0.096) and 0.016 ± 0.011 (*p* = 0.162); SEF95 changes were − 0.002 ± 0.002 Hz (*p* = 0.390) and − 0.002 ± 0.002 Hz (*p* = 0.326) for left and right hemispheres, respectively. However, the area under the curve of the power spectral (*p* < 0.001) and all frequency bands power (*p* < 0.001) showed a significant decrease over the time in both EEG. Minimal changes without clear pattern were observed in spectral slope during the study. BIS Index and SEF95 remained consistent during prolonged Trendelenburg positioning. The reduction in EEG singal power across 0–45 Hz caused a vertical shift in the power spectrum with minimal changes in its spectral profile. Facial tissue edema by positioning plays a primary role in the EEG signal power reduction.

**Supplementary Information:**

The online version contains supplementary material available at 10.1007/s10877-025-01403-x.

## Introduction

Cortical brain activity monitoring during anesthesia aims to reduce to decrease the likelihood of intraoperative awareness, and to help individualize anesthesia administration. It assesses the cerebral repercussions of intraoperative events to reduce the risk of neurological complications [1–8]. It is most commonly measured through level of anesthesia (LOA) monitors that give a scaleless index between 0 and 100 to express the anesthetic level. This index is obtained from mathematical algorithms registered by electrodes placed onto the patient´s forehead. Most of these algorithms are company-owned and not disclosed to the public, while others have been recently published [9–12]. Among them, one of the most used and studied algorithm is the bispectral (BIS) index, implemented by the BIS Vista™ monitor (Medtronic Inc., Minneapolis, MN, USA) [13, 14]. 

LOA monitors estimations can be affected by several factors such as the environment, patient clinical status, anesthetic drug dosing regime and surgical requirements. Electric devices such as convective air warming systems, pacemakers, cardiopulmonary bypass machines, endoscopic devices (shaver) are known to artifact BIS Index measurements [15, 16]. Electrocardiogram (ECG) contamination can interfere BIS Index [17]; hypoglycemia, hypo or hypercapnia and hypothermia can also alter BIS Index calculations [14, 16]. Drugs such as ketamine or nitrous oxide can also affect it [15, 18–22]. In addition, patient position during surgery may also substantially impact BIS results, especially when it modifies the head´s relative position to the chest [23, 24]. 

Robot-Assisted Radical Prostatectomy (RARP) is a relatively recent technique with increasing popularity. Advantages include less invasiveness, better pain management and reduced length of stay [25]. Anesthetic management for RARP can be challenging because the patient is positioned in steep Trendelenburg, and a pneumoperitoneum is established. This makes continuous muscular blockade advisable, but increases the chance of not recognising potential awareness. Homeostasis, ventilation, intraocular pressure and venous and lymphatic drainage from the cervical and cephalic territory can be impaired. Additionally, soft-tissue edema (involving the tongue, eyelids, conjunctiva and scalp) is common, raising concerns about the consistency of the BIS measurements over time, as soft tissue edema increases in the patient´s forehead.

This study aims to describe EEG evolution in time in the BIS Vista™ monitor and conventional EEG electrodes in patients in steep Trendelenburg position to assess the accuracy and consistency of the LOA monitor.

## Methods

### Ethical approval

for this study (Ethical Committee EO18/7) was provided by the Ethical Committee of Navarre, Navarre´s Health Department, Spain (Chairperson Prof Jesús M. Arteaga Coloma) on July 20th 2018. Prospective observational study (CUN-SUG-2018-01) including male patients undergoing RARP at *Clínica Universidad de Navarra* from October 2018 to November 2021 (Principal investigator: Iñigo Rubio). Inclusion criteria were male patients undergoing elective RARP employing *da Vinci X* (Intuitive Surgical, Sunnyvale, CA, USA)who accepted to participate and signed the informed consent. Exclusion criteria were refusal to participate; not using BIS Vista™ monitor and preexistent relevant neurological diseases that could alter the recording, such as stroke, epilepsy, drug abuse, neuroleptic or benzodiazepine treatment, neuromuscular disorders, or moderate-to-severe vascular disease.

The anaesthesia protocol included maintenance with sevoflurane and opioids, targeted to obtain a DSA with predominating alpha and delta activity, with low or suppression of activity in the beta band, maintaining SEF95 between 10 and 14 Hz (Appendix 1) [26]. All patients were monitored with the BIS Vista™ monitor using a bilateral sensor. Raw data including SEF95 (frequency encompassing 95% of the total EEG power), BIS Index, ASYM, TOTPOW, EMG and EEG (EEG-Bis) were obtained by exporting the signal from an external storage device via a USB Type A port on the back of the monitor. Complementary, a 4-channel EEG (EEG-BrVis) was set up to perform discontinuous EEG recordings using the BrainVision Recorder^®^ (GmbH, Gilching, Bayern, Germany) software; the amplifiers were from BrainVision BrainAmp^®^ (GmbH, Gilching, Bayern, Germany), all compatible with Windows 7 OS. The electrodes placed were Technomed medical accessories (Technomed Europe, Maastrich-Airport, Netherlands). The electrodes (corks) were positioned at the 10/20 system points F3, F4, P3, P4, all referenced to Pz in order to be able to perform transverse (right-left) and longitudinal (frontal-parietal) montages.

We simultaneously and synchronously monitored the parameters obtained with the BIS Vista™ monitor and the EEG with the 4-electrode EEG setup. Continuous recordings were made of the parameters obtained from the BIS Vista™ monitor and discontinuous recordings (about 5 min length) of the EEG-BrVis at each of the defined times. Subsequently, 120-second periods free of artifacts were selected and extracted for each of the moments in order to analyze and compare data for the same time point (See Appendix 2 for BIS Vista™ and EEG-BrVis data treatment).

We performed a baseline recording, which took place after anesthetic induction, surgical cleaning and sterile field placement, but prior to the Trendelenburg positioning and the start of the surgical intervention. The second recording took place 30 min after the positioning in steep Trendelenburg, and after that we performed a recording every 60 min until the end of the procedure (Supplementary Digital Material: Supplementary Fig. 1).

Operating table`s angulation was measured using a digital inclinometer (Digi-pas DWL80E, Digipas Technologies Inc., Irvine, USA). We defined as steep Trendelenburg angulations greater than 30º with respect to the floor.

In addition to the data exported, heart rate (HR), blood pressure, pulse oximetry (SpO_2_), capnography (ETCO_2_) and MAC were recorded to adjust for possible confounding factors.

Due to the exploratory nature of this study, no formal statistical sample size estimation was performed. A target of at least 15 patients was considered reasonable based on feasibility and previous experience.

Patient characteristics were summarized using means and standard deviations; medians and interquartile ranges; and counts and percentages. A data analysis and statistical plan was written after the data were accessed. To compute the area under the curve of the spectral power (PSD-AUC), the integral of the power spectral density was evaluated within the defined frequency range. The spectral slope (X) (power-law-slope) was estimated by fitting a linear function to the power spectrum represented in double-logarithmic units. In addition to the slope, the intercept of the fitted line with the power axis (b) (power-law-intercept) was also determined. Linear mixed-effects models were used in order to take into account repeatedly measured outcomes in the same patients. Time was included as a continuous variable for the calculation of coefficients when analyzing the trend of powers over time. Normality of residuals was assessed using QQ plots. Statistical significance was set at *P* < 0.05. Statistical analyses were performed using Stata 14 software (StataCorp. 2015. Stata Statistical Software: Release 14. College Station, TX, StataCorp LP).

## Results

A total of 20 patients were recruited. No patient was excluded for clinical reasons (problems related to the anesthetic or surgical procedure) or problems regarding EEG-BrVis data collection. Due to data exporting problems with the BIS Vista™ monitor, 2 patients were excluded. Therefore, 18 patients were included in the final analysis. Demographic data are shown in Supplementary Digital Material: Supplementary Table 1.

There was a high variability in the surgical procedure duration, with a mean ± SD duration of 269 ± 69 min. In 18 cases, the steep Trendelenburg positioning lasted more than 90 min; in 16 patients more than 150 min; and in 10 patients more than 210 min. The Trendelenburg angulation range was between 32º and 41º. There was no change in the table angulation once the steep Trendelenburg was set until the end of the procedure.

The BIS Index showed no significant differences in either cerebral hemisphere when comparing the baseline values with later time endpoints (Table 1). Statistical analyses did not detect any significant trend in the BIS Index with increasing time in steep Trendelenburg position (Table 2).

**Table 1:** Descriptive data on the evolution of the BIS index and SEF95 and the relationship of the baseline data with the different moments of steep Trendelenburg monitoring

Parameter (Units)		Time since steep Trendelenburg positioning				
		Basal (*n* = 18)	30 min (*n* = 18)	90 min (*n* = 18)	150 min (*n* = 16)	210 min (*n* = 10)
BIS Index	Left Hemisphere, mean ± SD	44.5 ± 9.9	43.1 ± 5.7	43.5 ± 6.3	47.4 ± 9.0	45.8 ± 8.8
	Coefficient ± SE	Ref.	-1.4 ± 2.5	-1.0 ± 2.6	3.2 ± 2.8	2.1 ± 2.4
	*p* value (compared to basal)	Ref.	0.57	0.71	0.26	0.40
	Right Hemisphere, mean ± SD	46.6 ± 13.3	42.7 ± 4.9	44.8 ± 5.9	47.5 ± 7.4	46.8 ± 8.3
	Coefficient ± SE	Ref.	-3.8 ± 3.2	-1.7 ± 3.3	1.4 ± 3.0	1.2 ± 2.9
	*p* value (compared to basal)	Ref.	0.23	0.60	0.63	0.68
SEF95 (Hz)	Left Hemisphere, mean ± SD	15.7 ± 2.0	14.8 ± 1.3	14.7 ± 1.4	15.2 ± 2.2	15.1 ± 1.2
	Coefficient ± SE	Ref.	-0.9 ± 0.4	-1.0 ± 0.4	-0.5 ± 0.6	-0.8 ± 0.4
	*p* value (compared to basal)	Ref.	0.02	0.01	0.37	0.06
	Right Hemisphere, mean ± SD	16 ± 1.9	15 ± 1.3	15.1 ± 1.4	15.4 ± 1.9	15.3 ± 1.8
	Coefficient ± SE	Ref.	-1.0 ± 0.4	-0.9 ± 0.4	-0.6 ± 0.5	-0.8 ± 0.4
	*p* value (compared to basal)	Ref.	< 0.01	0.01	0.21	0.05

SD: Standard Deviation. SE: Standard error. The coefficients indicate the estimated average change in the variable relative to baseline

Baseline SEF95 values showed a statistically decrease when compared with 30 and 90 min after the steep Trendelenburg but disappeared or attenuated for later times (150 and 210 min) (Table 1). The magnitude of these changes, although statistically significant in some points was restricted to variations of 1 Hz. Accordingly, the analysis of changes over time of the SEF95 did not show any significant trend in the SEF95 value with increasing time in steep Trendelenburg position (Table 2).

**Table 2:** Trends of BIS index and SEF95 during steep Trendelenburg position over time

		Coefficient ± SE	*P* value
BIS Index	Left Hemisphere	0.017 ± 0.010	0.096
	Right Hemisphere	0.016 ± 0.011	0.162
SEF95	Left Hemisphere	-0.002 ± 0.002	0.390
	Right Hemisphere	-0.002 ± 0.002	0.326

SE: Standard error

**Fig. 1:**
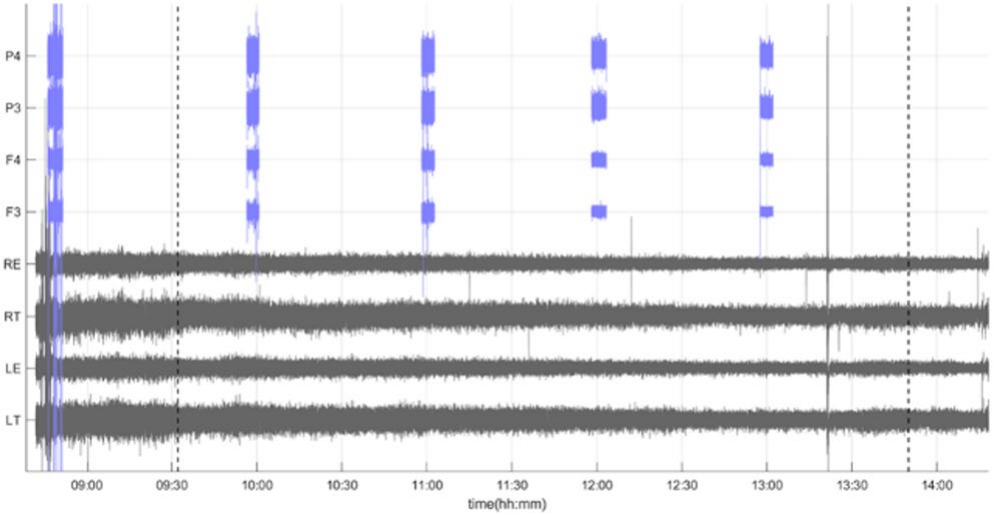
Simultaneous and synchronised monitoring of one of the included cases. In grey we have the continuous signal of the EEG-BIS recording with their respective electrodes, and in blue, synchronously placed, the discontinuous recordings of the EEG-BrVis montage at each of the electrodes. The vertical dashed lines indicate the beginning and end of the steep Trendelenburg

**Fig. 2:**
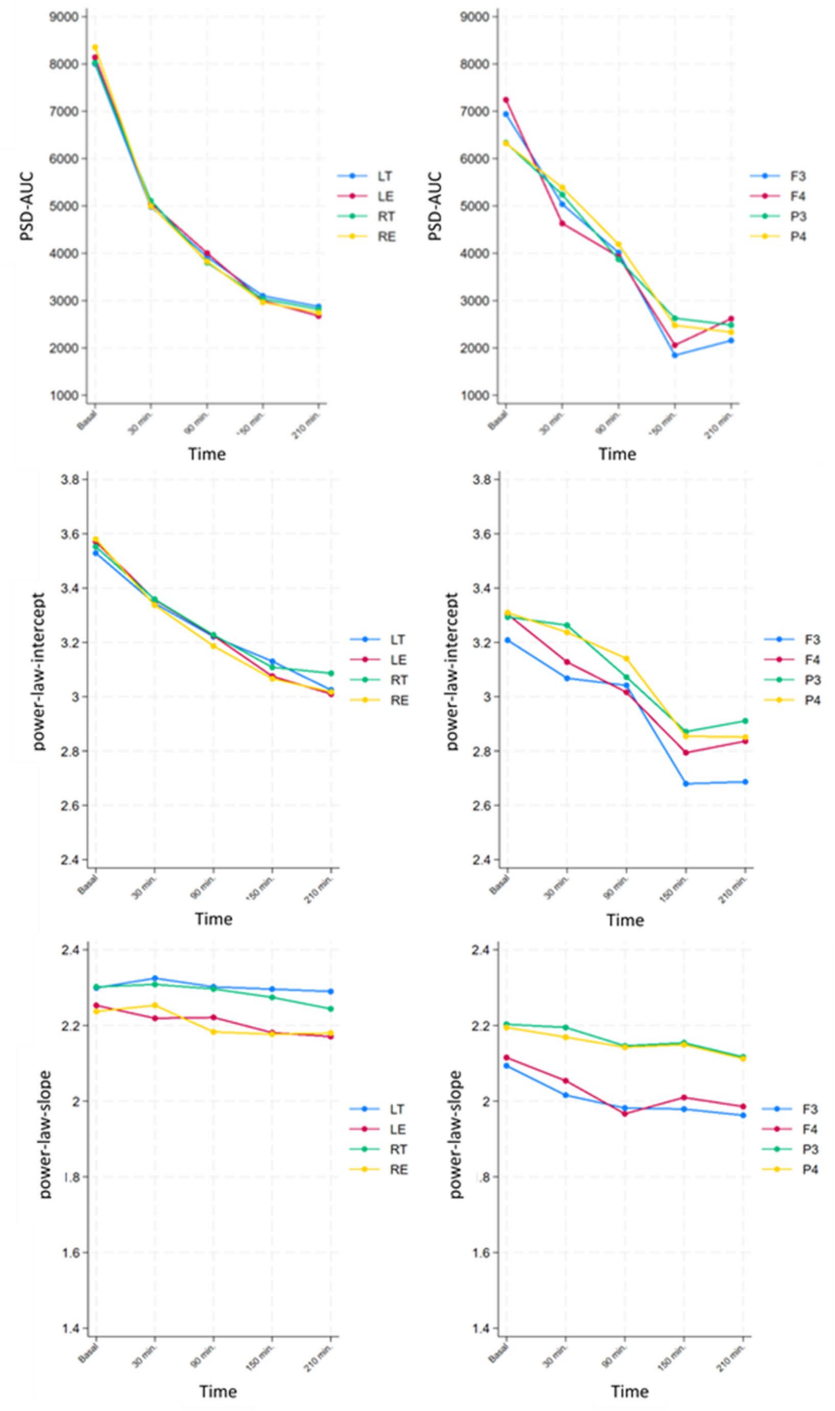
Graphical representation of expected values for PSD-AUC, power-law-intercept and power-law-slope from both EEG montages in the differents moments of the study. PSD-AUC: Area under the curve of the spectral power. Power-law-Intercept: The intercept of the fitted line with the power axis. Power-law-Slope: The slope of a power law fit of the spectral profile LT, LE, RT and RE: EEGBis electrodes. F3, F4, P3 and P4: EEGBrVis electrodes

Regarding the EEG data, our patients experimented a decrease in the EEG signal power with increasing time in the steep Trendelenburg. Figure 1 shows a visual example of the progressive decrease for a representative subject, with the two montages (EEG-BIS and EEG-BrVis) superimposed and synchronized. To explore this signal decrease we analyzed the area under the curve of the spectral power (PSD-AUC), the intercept of a power law fit of the spectral profile (power-law-intercept) and frecuency bands power (Appendix 2). We observed a statistically significant decrease in PSD-AUC (Fig. 2A) and power-law-intercept (Fig. 2B) from baseline to the different moments (Supplementary Table 2) and with increasing time in the steep Trendelenburg position (Table 3) from both EEG-BIS and EEG-BrVis montages. Consistent with these results, a significant reduction in the signal power was observed for every frequency band, in every electrode and time points, from baseline to the different moments (Supplementary Table 2) and with increasing time in the steep Trendelenburg position (Table 3).

**Fig. 3:**
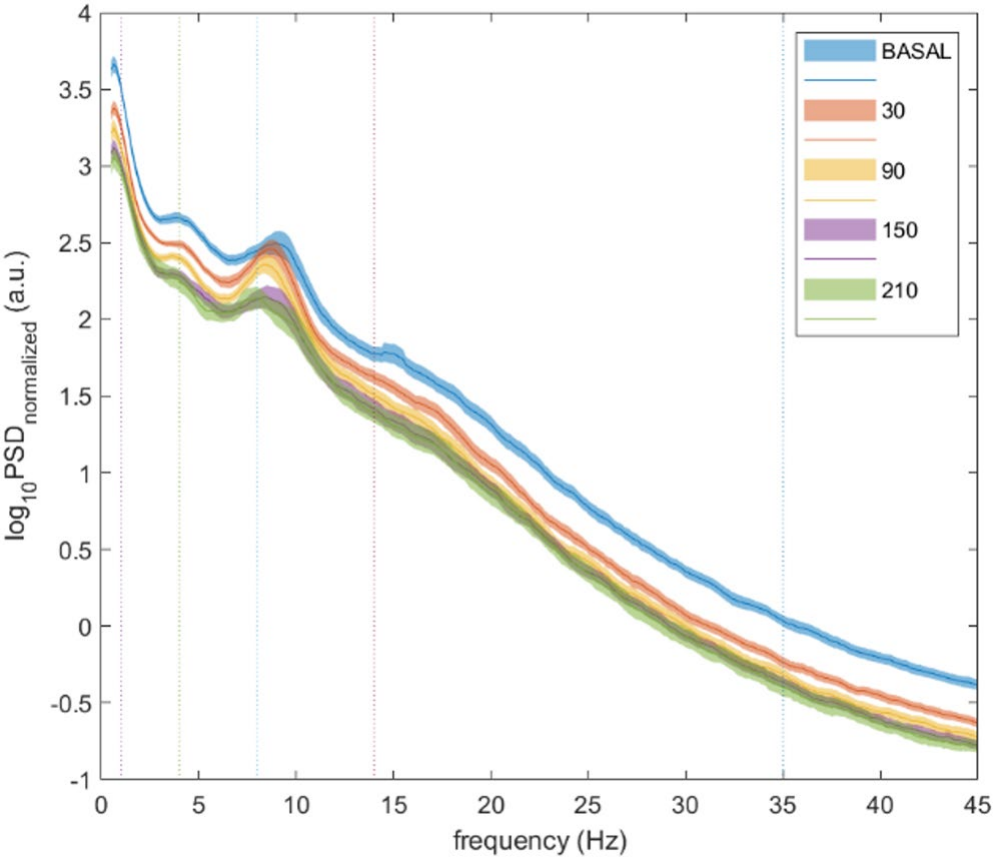
Graphical representation of the frequency spectra of each wave at each of the specific times (colours) in a patient in the study. In blue, power spectrum at the baseline recording; in orange, power spectrum at the recording 30 min after Trendelenburg; in yellow, power spectrum at the recording 90 min after Trendelenburg; in purple, power spectrum at the recording 150 min after Trendelenburg; in green, power spectrum at the recording 210 min after Trendelenburg

Figure 3 shows the mean (and confidence interval) semi-logarithmic plot of the normalized power from the EEG-BIS LT contact of the 18 patients evaluated at baseline and 30, 90, 150, and 210 min after steep Trendelenburg positioning. With increasing time in the steep Trendelenburg position, a homogeneous power decrease in cortical brain activity can be observed. Interestingly, this decrease seems to affect equally the whole set of frequencies analyzed (from 0 to 45 Hz) and results in a progressive vertical shift of the spectral profile without apparently altering the shape of the PSD or the power balance between bands.

To explore if the progressive vertical shift of the spectral profile associated alteration in the shape of the PSD we analyzed the changes in the slope of a power law fit of the spectral profile (power-law-slope) (Appendix 2). We did not find a consistent trend in the evolution of the spectral slope (Fig. 2C) when analyzed data from baseline to the different moments (Supplementary Table 2) nor in the trend over (Table 3) in both EEG montages. Although some electrodes showed apparent changes at some of the moments analyzed, the magnitude of this change was restricted to slight changes in signal power (Table 3, Supplementary Table 2).

Changes of hemodynamic, oximetry and capnography parameters from baseline to 210 min after Trendelenburg were also registered and analyzed. Differences between baseline SBP values and 30 min values were statistically significant, but without clinical relevance. No significant adjustements of sevoflurane were required to maintain a stable SEF95 in our patients (MAC values). No other significant changes were observed. These data are specified in Supplementary Digital Material (Supplementary Table 3).

**Table 3:** Time trend analysis of each of the EEG bands

			PSD-AUC	Power-law-intercept	Beta	Alpha	Theta	Delta	Power-law-slope
EEG-BIS	RT	Coeff. ±SE	-23.21 ± 1.32	-2.3 × 10^− 3^ ±2 × 10^− 4^	-0.06 ± 0.01	-0.69 ± 0.12	-0.86 ± 0.68	-2.61 ± 0.26	-2.7 × 10^− 4^ ±1.6 × 10^− 4^
		*p* value	< 0.001	< 0.001	< 0.001	< 0.001	< 0.001	< 0.001	0.098
	RE	Coeff. ±SE	-24.96 ± 1.52	-2.6 × 10^− 3^ ±3 × 10^− 4^	-0.09 ± 0.02	-0.61 ± 0.10	-0.86 ± 0.08	-2.96 ± 0.27	-3.9 × 10^− 4^ ±1.5 × 10^− 4^
		*p* value	< 0.001	< 0.001	< 0.001	< 0.001	< 0.001	< 0.001	0.01
	LT	Coeff. ±SE	-22.72 ± 1.41	-2.3 × 10^− 3^ ±3 × 10^− 4^	-0.05 ± 0.01	-0.65 ± 0.11	-0.90 ± 0.09	-2.57 ± 0.27	-9.6 × 10^− 5^ ±9.2 × 10^− 5^
		*p* value	< 0.001	< 0.001	< 0.001	< 0.001	< 0.001	< 0.001	0.299
	LE	Coeff. ±SE	-24.31 ± 1.53	-2.6 × 10^− 3^ ±2 × 10^− 4^	-0.08 ± 0.01	-0.61 ± 0.09	-0.89 ± 0.09	-2.87 ± 0.27	-3.8 × 10^− 4^ ± 1.5 × 10^− 4^
		*p* value	< 0.001	< 0.001	< 0.001	< 0.001	< 0.001	< 0.001	0.012
EEG-BrVis	F4	Coeff. ±SE	-22.76 ± 4.49	-2.5 × 10^− 3^ ±5 × 10^− 4^	-0.06 ± 0.01	-0.37 ± 0.09	-0.61 ± 0.12	-2.03 ± 0.42	-5.9 × 10^− 4^ ±1.8 × 10^− 4^
		*p* value	< 0.001	< 0.001	< 0.001	< 0.001	< 0.001	< 0.001	0.001
	P4	Coeff. ±SE	-20.88 ± 2.82	-2.5 × 10^− 3^ ±3 × 10^− 4^	-0.04 ± 0.01	-0.51 ± 0.12	-0.68 ± 0.11	-1.97 ± 0.32	-3.3 × 10^− 4^ ±1.6 × 10^− 4^
		*p* value	< 0.001	< 0.001	< 0.001	< 0.001	< 0.001	< 0.001	0.035
	F3	Coeff. ±SE	-24.57 ± 5.19	-2.7 × 10^− 3^ ±5 × 10^− 4^	-0.04 ± 0.01	-0.25 ± 0.41	-0.52 ± 0.1	-2.14 ± 0.38	-5.6 × 10^− 4^ ±2.1 × 10^− 4^
		*p* value	< 0.001	< 0.001	< 0.001	< 0.001	< 0.001	< 0.001	0.007
	P3	Coeff. ±SE	-19.81 ± 2.81	-2.3 × 10^− 3^ ±3 × 10^− 4^	-0.04 ± 0.01	-0.54 ± 0.13	-0.71 ± 0.12	-1.92 ± 0.34	-3.9 × 10^− 4^ ±1.3 × 10^− 4^
		*p* value	< 0.001	< 0.001	< 0.001	< 0.001	< 0.001	< 0.001	0.003

Coeff.: Coefficient. SE: Standard error. PSD-AUC: Area under the curve of the spectral power. Power-law-Intercept: The intercept of the fitted line with the power axis. Power-law-Slope: Changes in the slope of a power law fit of the spectral profile. The coefficients represents the average change per unit of time

## Discussion

LOA monitoring with the BIS Vista™ can be influenced by environmental factors, clinical status or extreme positions. In our study, the BIS Index and SEF95 remained stable while a progressive decrease in EEG signal power was observed over time in patients in steep Trendelenburg positioning.

Kaki et al. registered a 6.33-point median increase in BIS Index when steep Trendelenburg was established and a 3.5-point median decrease in BIS Index when reverse-Trendelenburg was established [23]. Lee et al. found a 10–13 points mean decrease in BIS Index when Fowler’s position was established after anesthetic induction [24]. Both studies attributed these findings to changes in cerebral perfusion [27, 28], a controversial hypothesis [29, 30]. 

In contrast, our study found that the BIS Index was consistently stable over time in steep Trendelenburg. No significant differences were noted between baseline and subsequent time points in steep Trendelenburg, or when calculating the change over time. This difference likely stems from monitoring duration. Unlike earlier studies focused on the 15 min after anesthetic induction (a period marked by drug-induced hemodynamic effects), we monitored most patients for over 210 min, ensuring a 15 min period in a neutral position was respected prior to steep Trendelenburg [23, 24]. Hemodynamic data were analyzed to assess potential relationships.

For SEF95, we observed a statistically significant reduction in the 30 and 90 min post-Trendelenburg compared to baseline. This reduction disappeared at 150 min and barely returned at 210 min. Although consistent over both hemispheres, the reduction got going from slight changes in signal, with a maximum difference in mean values of 1 Hz. When analyzing the significance of the change, we did not detect any significant trend. These results suggest that SEF95 remains stable during prolonged steep Trendelenburg, although further studies are needed to confirm this. Although the study protocol indicated adjusting the sevoflurane MAC for a SEF95 between 10 and 14 Hz, the anesthesiologists in charge of the cases felt comfortable with a SEF95 between 14 and 16 Hz, without making significant changes to the MAC to comply with the study protocol.

We have observed an overall decreased recorded EEG signal power that is statistically significant and in the same direction at virtually all electrodes in both EEG-BIS and EEG-BrVis montages (Figs. 1, 2 and 3; Table 3 and Supplementary Table 2). We observed that steep Trendelenburg positioning leads to a considerable loss of spectral density, as reflected by the decrease in the PSD-AUC. When comparing this decline over time during extreme Trendelenburg, we found that the greatest power loss occurs within the first periods analyzed, with subsequent measurements showing an attenuation of this loss (Fig. 2A). This fact could be consistent with a decrease in EEG signal secondary to tissue congestion or edema. Furthermore, when comparing the decrease in power between the BIS monitor sensor (EEG-BIS) and the 4-electrode EEG setup (EEG-BrVis), we observed that the loss of power is greater with the BIS sensor than subdermal corkscrew electrodes (Fig. 2A). This difference in the magnitude of power loss may be due to the fact that the subdermal corkscrew electrodes used in the EEG-BrVis system are less exposed to tissue congestion or edema since they penetrate down to subcutaneous tissue.

When considering each frequency band separetely, with minor exceptions—specifically, minimal increases in the power of beta, alpha, and delta waves at electrodes P4 and F4 at 150 and 210 min post-Trendelenburg, likely attributable to the reduced number of recordings at final (*n* = 10) intervals—there is a decrease in the power of all frequencies and electrodes with increasing time in steep Trendelenburg position. All the changes (for every frequency band and electrode) are negative and statistically significant, confirming a progressive reduction of the power of the EEG, regardless of the frequency band, recording location, or EEG modality. Interestingly, this power reduction affects equally the whole set of frequencies (0–45 Hz), resulting in a vertical shift of the power spectrum without affecting the spectral profile. The change also impacted the interpretation of the density spectral array (DSA), with reduced warmth in tone intensity potentially misinterpreted as deeper anesthesia.

To further evaluate the pattern of this decline in signal power, we analyzed the changes in slope across the different spectrograms of each patient at each of the time points assessed during steep Trendelenburg (power-law-slope). Although some electrodes showed apparent changes at some of the moments analyzed, the magnitude of this change were minimal, inconsistent, and lacked a discernible pattern (Table 3, Supplementary Table 2). Despite the documented increases in intracranial pressure, and the demonstrated facial and laryngeal edema associated with the steep Trendelenburg positioning, there is no enough evidence demonstrating the routine development of cerebral edema [29, 31, 32]. Based on our results, a reduction of the overall power of the EEG that suggests vascular congestion or slope tissue edema as a more plausible explanation for the observed changes than cerebral edema [33]. Nevertheless, we cannot exclude a potential contribution from cerebral edema, which may play a minor role in the changes observed in spectral slope (power-law-slope) over time in steep Trendelenburg. This does not affect our assertion that both the BIS index and SEF95 remain stable and reliable.

The development of facial slope tissue edema, in contrast to the relative preservation of cerebral edema, is attributed to differences in intra- and extracranial circulation during steep Trendelenburg positioning [32, 34]. Gravitational shifts also influence blood flow in the internal and external carotid arteries [35]. While facial edema is among the most frequently reported adverse effects of prolonged steep Trendelenburg positioning [36], cerebral blood flow velocity seem to remain stable [30, 32]. 

Regarding hemodynamics, we found no significant changes in HR and MAC. SBP showed a significant increase 30 min into steep Trendelenburg, likely due to surgical onset or position-related preload changes. EtCO2 showed significant increases at 150 and 210 min, coinciding with pneumoperitoneum release and the conclusion of surgery, including specimen extraction and wound closure. Although statistically significant, this increases are clinically irrelevant (only 2.1 mmHg compared to basal); this values have remained within normal range throughout the whole procedure.

Limitations include the exploratory nature of the study and the homogeneus patient population (sex, age and surgery type). In addition, no formal sample size estimation was conducted. The sample size was based on feasibility and previous experience, and finally determined by the availability of patients data in a context of clinical practice. Finally, anesthesiologists should have increased the MAC of sevoflurane to adjust the SEF between 10 and 14 Hz during the study, as indicated in the protocol. Future research should confirm these findings in diverse scenarios to validate their discriminatory capacity.

## Conclusions

In conclusion, we found no significant differences in the evolution of EEG-BIS and EEG-BrVis during prolonged steep Trendelenburg. Both BIS Index and SEF95 remained stable. The global signal power decrease observed is uniform and with minimal changes in its profile. Facial tissue edema and tissue congestion by possitioning appear to play a primary role in the reduction of EEG signal power, although a contribution from cerebral edema cannot be excluded.

## Appendix 1: anaesthetic management of robotic assisted radical prostatectomy

*Monitoring*.

- ECG, Non invasive Blood Pressure, SpO_2_, Capnography, Temperature.
- Arterial line (invasive blood pressure).
- Train of four (TOF).
- BIS bilateral.
- 5-Channel EEG (depending on Neurophysiology): F3-F4-C3-C4-CZ-Ref.

*Induction*.

- *NO Benzodiazepines*.
- Fentanyl IV: 1-1.5 mcg/kg.
- Propofol IV: 1.5–2.5 mg/kg.
- (or Etomidate IV: 0.2–0.4 mg/kg)
- Rocuronium IV: 0.6–1.2 mg/kg.

*Maintenance*.

- Sevoflurane: MAC 0.8–1.2 (Target SEF 10–14 Hz).
- Rocuronium IV (Continuous infusion: 0.3–0.4 mg/kg/h.
- (Optional) Remifentanil IV (Continuos infusion, 20/40 mcg/ml): TCI 0.5-4 mcg/ml.
- (Optional) Fentanyl IV bolus: 50–100 mcg. Max. 0.5–0.7 mcg/kg.
- (Optional) IV analgesia: Acetaminophen, metamizole, morphine.

*Eduction*.

- STOP Rocuronium continuous infusion after completion of urethral suture.
- Neuromuscular blockade verification (TOF):
- If TOF = 4 & TOF > 90%: No sugammadex needed.
- If TOF = 3, or 4 & TOF < 90%: Sugammadex 2 mg/kg.
- If TOF = 2: Sugammadex 4 mg/kg.
- If TOF = 1: Sugammadex 8 mg/kg.
- If TOF = 0: Sugammadex 16 mg/kg.

*In obese patients, dosing is based on actual weight.

- Sugammadex administration and BIS-EEG recording after skin suture.
- Stop sevoflurane and remifentanil continuous infusion.
- Analgesia.
- Extubation (optional).

*BIS-EEG recording*.

- BIS-EEG recording parameters:
- MAC: 0.9-1 (adjusted to patient’s age) (Target SEF95: 10–14 Hz).
- TCI remifentanil: 2 mcg/ml.
- BIS-EEG recording moments:
  - Baseline after induction.
  - 30 min after steep Trendelemburg.
  - 90-150-210 min after steep Trendelemburg.
  - Just before reseting operating table back to initial position (0º).
  - 15–30 min after reseting operating table back to initial position (0º).
  - During sugammadex administration.

*During data recording (2 min period recording EEG), try not to use electric scalpel.

## Appendix 2

We used custom MatlabTM scripts developed by the research team were then used to import and process the files transferred from the BIS VISTATM for further analysis. The SEF95 and BIS index have a sampling rate of 1 Hz, while EEG data are sampled at 128 Hz. EEG signals through the BrainAmp amplifier (EEG-BrVis) signals were amplified and filtered from 0.3 to 1,000 Hz, sampled at 2,000 Hz with a resolution of 0.1 µV and a resolution of 0.1 µV, and stored for further analysis using MatlabTM.

For data analysis, 120 s blocks of the EEG-BIS signal and the EEG-BrVis signal in X, Y, Z were selected for analysis. Hereafter we estimated the BIS index and SEF95 means within the 2-minute blocks (i.e. 120 samples) for the left and right sides. For analysis of the EEG data (both EEG-BIS and EEG-BrVis), we followed the approach described by our previous investigations by Martínez-Simón et al. [37, 38] We estimated the spectral content of EEG signals between 0 and 45 Hz using a multitaper spectral analysis implemented with the mtspectrumc function of the Chronux applications using an 8-percent window. Chronux applications using an 8-second window overlapped at 75% with tapper parameters TW = 4 and K = 9, where TW is the time-bandwidth product and K is the number of tapes.

We decided to carry out the normalisation of the spectral values by dividing them by the spectrum mean (estimated over the whole set of spectra for each channel and subject, but for each channel and subject, but considering the X blocks) and multiplying by 100. Finally, the total power in each band [delta (1–4 Hz), theta (4–8 Hz), alpha (8–14 Hz), beta (14–35 Hz) and gamma (35–45 Hz)] was estimated by averaging the normalised values within the corresponding ranges.

To compute the area under the curve of the spectral power (PSD-AUC), the integral of the power spectral density was evaluated within the defined frequency range. The spectral slope (X) (power-law-slope) was estimated by fitting a linear function to the power spectrum represented in double-logarithmic units. In addition to the slope, the intercept of the fitted line with the power axis (b) (power-law-intercept) was also determined.

## Supplementary Information

Below is the link to the electronic supplementary material.

Supplementary Material 1

Supplementary Material 2

Supplementary Material 3

Supplementary Material 4

## Data Availability

No datasets were generated or analysed during the current study.

## Declarations

### Finding

The authors declare that no funds, grants, or other support were received during the preparation of this manuscript.

### Implication statement

The uniform reduction in EEG activity and changes observed in the Density Spectral Array (DSA) during Trendelenburg position contrast with the stability of BIS Index and SEF95. These results suggest that BIS Index and SEF95 remain consistent. The observed EEG power reduction may be attributed to subcutaneous edema.
