# Supplementary material for: Effects of sustained Trendelenburg position on the spectral signatures of the EEG: implications for the consistency of the level of anesthesia, an observational study
Source: J Clin Monit Comput. 2025 Dec 22;40(2):323–32. doi: 10.1007/s10877-025-01403-x (PMC13053590; doi:10.1007/s10877-025-01403-x)
Supplement: Supplementary file 2 — Supplementary Material 2 [file 10877_2025_1403_MOESM2_ESM.docx]

**SUPPLEMENTARY FIGURE LEGEND**

Supplementary Figure 1. Summary of each of the times at which EEG recordings have been registered.
