# Supplementary material for: Effects of sustained Trendelenburg position on the spectral signatures of the EEG: implications for the consistency of the level of anesthesia, an observational study
Source: J Clin Monit Comput. 2025 Dec 22;40(2):323–32. doi: 10.1007/s10877-025-01403-x (PMC13053590; doi:10.1007/s10877-025-01403-x)
Supplement: Supplementary file 3 — Supplementary Material 3 [file 10877_2025_1403_MOESM3_ESM.docx]

**SUPPLEMENTARY TABLES**

Supplementary Table 1. Demographic data.

| Demographic | |
| --- | --- |
| N | 18 |
| Age (years), median (IQR) | 66 (55 a 79) |
| BMI, kg⋅ m^-2^, mean (SD) | 26.3 (2.6) |
| Comorbidities | |
| Hypertension, n (%) | 9 (50) |
| Dyslipemia, n (%) | 5 (28) |
| Diabetes mellitus, n (%) | 1 (6) |
| Ischemic heart disease, n (%) | 4 (22) |
| METS, median (IQR) | 3 (2.0 a 4.0) |
| Treatment | |
| Anti- hypertensive, n (%) | 5 (28) |
| Statins, n (%) | 7 (39) |
| Alpha- blockers, n (%) | 3 (17) |
| Antiaggregants, n (%) | 2 (11) |
| Anticoagulants, n (%) | 0 (0) |

BMI: Body mass index. IQR: Interquartile range. METS: Metabolic equivalents. SD: Standard deviation.

Supplementary Table 2. Numerical description of the evolution of the different frequency range bands (beta, alpha, theta and delta) and relationship of the baseline data with the different moments of steep Trendelenburg monitoring.

| Wave | Elect. |  | Time since Trendelenburg positioning | | | | |
| --- | --- | --- | --- | --- | --- | --- | --- |
|  |  |  | Basal (n=18) | 30min (n=18) | 90min (n=18) | 150min (n=16) | 210min (n=10) |
| Beta (12-40 Hz) | BIS_RT | Mean (SD) | 19.0 (9.1) | 10.9 (5.0) | 8.5 (4.0) | 7.0 (3.5) | 6.9 (4.0) |
|  |  | Coeff. (SD) | Ref. | -8.1 (1.5) | -10.6 (1.6) | -12.3 (1.8) | -13.6 (2.1) |
|  |  | *p* value | Ref. | <0.001 | <0.001 | <0.001 | <0.001 |
|  | BIS_RE | Mean (SD) | 29.2 (22.0) | 13.6 (7.8) | 10.8 (6.4) | 8.5 (5.1) | 8.5 (6.3) |
|  |  | Coeff. (SD) | Ref. | -15.6 (4.6) | -18.4 (4.9) | -21.2 (4.94) | -21.9 (4.6) |
|  |  | *p* value | Ref. | <0.001 | <0.001 | <0.001 | <0.001 |
|  | BIS_LT | Mean (SD) | 18.2(8.6) | 10.6 (5.0) | 8.2 (4.1) | 7.0 (3.9) | 6.7 (4.2) |
|  |  | Coeff. (SD) | Ref. | -7.6 (1.4) | -10 (1.5) | -11.5 (1.7) | -12.9 (2) |
|  |  | *p* value | Ref. | <0.001 | <0.001 | <0.001 | <0.001 |
|  | BIS_LE | Mean (SD) | 24.5 (13.4) | 14.1 (7.4) | 10.4 (5.7) | 8.7 (5.3) | 8.5 (5.9) |
|  |  | Coeff. (SD) | Ref. | -10.5 (2) | -14.2 (2.3) | -16.4 (2.5) | -18.4 (3.1) |
|  |  | *p* value | Ref. | <0.001 | <0.001 | <0.001 | <0.001 |
|  | BrVis_F4 | Mean (SD) | 17.5 (14.9) | 11.2 (8.3) | 8.9 (7.0) | 5.6 (4.5) | 7.0 (8.2) |
|  |  | Coeff. (SD) | Ref. | -6.3 (2) | -8.5 (2.6) | -11.7 (2.8) | -11.9 (3.4) |
|  |  | *p* value | Ref. | 0.002 | <0.001 | <0.001 | <0.001 |
|  | BrVis_P4 | Mean (SD) | 11.6 (8.0) | 8.2 (3.8) | 6.3 (3.5) | 4.3 (2.9) | 4.0 (2.1) |
|  |  | Coeff. (SD) | Ref. | -3.4 (1.3) | -5.2 (1.3) | -7.1 (1.4) | -7.6 (1.7) |
|  |  | *p* value | Ref. | 0.008 | <0.001 | <0.001 | <0.001 |
|  | BrVis_F3 | Mean (SD) | 14.2 (11.0) | 9.0 (5.9) | 7.7 (6.1) | 4.3 (3.5) | 4.7 (6.6) |
|  |  | Coeff. (SD) | Ref. | -5.2 (1.9) | -6.5 (1.9) | -9.5 (2.1) | -8.5 (1.9) |
|  |  | *p* value | Ref. | 0.006 | 0.001 | <0.001 | <0.001 |
|  | BrVis_P3 | Mean (SD) | 12.7 (8.6) | 8.6 (4.6) | 6.7 (3.5) | 4.9 (3.0) | 4.5 (2.7) |
|  |  | Coeff. (SD) | Ref. | -4.1 (1.2) | -6.1 (1.4) | -7.9 (1.5) | -8.3 (1.8) |
|  |  | *p* value | Ref. | 0.001 | <0.001 | <0.001 | <0.001 |
| Alpha (8-12 Hz) | BIS_RT | Mean (SD) | 215.0 (136.7) | 149.8 (76.5) | 116.6 (63.5) | 83.9 (48.1) | 78.1 (48.8) |
|  |  | Coeff. (SD) | Ref. | -65.2 (26.8) | -98.4 (23.5) | -134.9 (30.5) | -146.9 (29.6) |
|  |  | *p* value | Ref. | 0.015 | <0.001 | <0.001 | <0.001 |
|  | BIS_RE | Mean (SD) | 181.6 (103.5) | 119.3 (62.3) | 87.5 (46.5) | 64.8 (36.6) | 60.9 (33.1) |
|  |  | Coeff. (SD) | Ref. | -62.3 (17.8) | -94 (18.6) | -119.3 (21.7) | -132.7 (23.9) |
|  |  | *p* value | Ref. | <0.001 | <0.001 | <0.001 | <0.001 |
|  | BIS_LT | Mean (SD) | 211.7 (133.2) | 151.1 (71.9) | 122.1 (64.6) | 89.1 (51.6) | 80.0 (47.2) |
|  |  | Coeff. (SD) | Ref. | -60.6 (27.9) | -89.6 (22.8) | -127.6 (30.2) | -139 (28.2) |
|  |  | *p* value | Ref. | 0.03 | <0.001 | <0.001 | <0.001 |
|  | BIS_LE | Mean (SD) | 188.4 (107.6) | 128.7 (57.8) | 98.7 (52.5) | 72.1 (41.6) | 66.5 (38.1) |
|  |  | Coeff. (SD) | Ref. | -59.7 (21) | -89.7 (17.7) | -121 (22.9) | -130.4 (22.9) |
|  |  | *p* value | Ref. | 0.005 | <0.001 | <0.001 | <0.001 |
|  | BrVis_F4 | Mean (SD) | 109.0 (95.7) | 86.3 (66.4) | 60.4 (45.5) | 44.3 (37.5) | 46.4 (36.4) |
|  |  | Coeff. (SD) | Ref. | -22.7 (10.4) | -48.6 (14.2) | -66.2 (16.7) | -76.1 (23.1) |
|  |  | *p* value | Ref. | 0.027 | <0.001 | <0.001 | <0.001 |
|  | BrVis_P4 | Mean (SD) | 154.6 (126.0) | 121.8 (82.4) | 94.2 (67.6) | 63.2 (48.5) | 58.5 (43.5) |
|  |  | Coeff. (SD) | Ref. | -32.8 (14.1) | -60.4 (16.8) | -91.8 (21.5) | -106.1 (27.9) |
|  |  | *p* value | Ref. | 0.02 | <0.001 | <0.001 | <0.001 |
|  | BrVis_F3 | Mean (SD) | 84.7 (55.3) | 66.3 (42.5) | 48.3 (34.6) | 30.8 (24.4) | 26.9 (24.0) |
|  |  | Coeff. (SD) | Ref. | -18.4 (7.2) | -36.4 (8.8) | -50.5 (8.9) | -47.3 (8.1) |
|  |  | *p* value | Ref. | 0.01 | <0.001 | <0.001 | <0.001 |
|  | BrVis_P3 | Mean (SD) | 168.0 (136.8) | 126.2 (81.6) | 102.0 (71.7) | 70.7 (53.5) | 60.7 (42.2) |
|  |  | Coeff. (SD) | Ref. | -41.8 (16.5) | -65.9 (19) | -98.6 (23.6) | -114.4 (30.4) |
|  |  | *p* value | Ref. | 0.011 | 0.001 | <0.001 | <0.001 |
| Theta (4-8 Hz) | BIS_RT | Mean (SD) | 321.0 (85.3) | 226.9 (48.0) | 176.1 (41.2) | 145.4 (52.9) | 137.1 (61.7) |
|  |  | Coeff. (SD) | Ref. | -94.1 (16.2) | -144.9 (18.5) | -174.8 (18.3) | -185.8 (14.9) |
|  |  | *p* value | Ref. | <0.001 | <0.001 | <0.001 | <0.001 |
|  | BIS_RE | Mean (SD) | 303.9 (93.0) | 210.8 (51.3) | 161.1 (42.5) | 135.6 (56.6) | 124.4 (53.9) |
|  |  | Coeff. (SD) | Ref. | -93.1 (15.5) | - 142.8 (18.7) | -170.7 (19.7) | -187.2 (18.5) |
|  |  | *p* value | Ref. | <0.001 | <0.001 | <0.001 | <0.001 |
|  | BIS_LT | Mean (SD) | 330.6 (96.2) | 231.1 (49.1) | 184.1 (46.8) | 148.7 (51.3) | 143.1 (61.4) |
|  |  | Coeff. (SD) | Ref. | -99.5 (18) | -146.5 (21.6) | -182.1 (21.2) | -196.5 (19.92) |
|  |  | *p* value | Ref. | <0.001 | <0.001 | <0.001 | <0.001 |
|  | BIS_LE | Mean (SD) | 308.2 (98.6) | 221.3 (57.2) | 170.6 (47.9) | 139.1 (54.4) | 129.8 (56.7) |
|  |  | Coeff. (SD) | Ref. | -86.8 (17.1) | -137.5 (20.5) | -173.3 (21.2) | -190.8 (20.6) |
|  |  | *p* value | Ref. | <0.001 | <0.001 | <0.001 | <0.001 |
|  | BrVis_F4 | Mean (SD) | 216.7 (126.6) | 169.5 (88.2) | 128.1 (65.6) | 92.4 (57.5) | 114.9 (95.4) |
|  |  | Coeff. (SD) | Ref. | -47.2 (15.1) | -88.7 (19.6) | -120.8 (20.8) | -121.2 (27.6) |
|  |  | *p* value | Ref. | 0.002 | <0.001 | <0.001 | <0.001 |
|  | BrVis_P4 | Mean (SD) | 273.8 (130.8) | 230.2 (89.6) | 180.7 (83.3) | 136.7 (64.3) | 138.1 (89.8) |
|  |  | Coeff. (SD) | Ref. | -43.5 (15.2) | -93 (21.7) | -128 (24.2) | -133.2 (21.8) |
|  |  | *p* value | Ref. | 0.004 | <0.001 | <0.001 | <0.001 |
|  | BrVis_F3 | Mean (SD) | 184.5 (122.2) | 140.5 (80.8) | 110.1 (61.6) | 72.9 (49.4) | 70.9 (71.9) |
|  |  | Coeff. (SD) | Ref. | -43.9 (15.5) | -74.3 (18.4) | -104.4 (20.9) | -104.1 (23.3) |
|  |  | *p* value | Ref. | 0.005 | <0.001 | <0.001 | <0.001 |
|  | BrVis_P3 | Mean (SD) | 289.1 (132.9) | 232.3 (82.5) | 189.6 (78.1) | 148.4 (68.7) | 140.3 (97.6) |
|  |  | Coeff. (SD) | Ref. | -56.7 (16.2) | -99.5 (23.8) | -138.3 (27.6) | -144.5 (24.2) |
|  |  | *p* value | Ref. | <0.001 | <0.001 | <0.001 | <0.001 |
| Delta (1-4 Hz) | BIS_RT | Mean (SD) | 945.0 (255.2) | 628.2 (170.6) | 464.9 (116.5) | 399.8 (134.4) | 399.6 (169.7) |
|  |  | Coeff. (SD) | Ref. | -316.8 (56.5) | -480.1 (56.9) | -552.2 (59.2) | -568.6 (54) |
|  |  | *p* value | Ref. | <0.001 | <0.001 | <0.001 | <0.001 |
|  | BIS_RE | Mean (SD) | 1004.6 (271.2) | 631.5 (193.2) | 468.7 (137.6) | 385.4 (133.1) | 377.5 (163.5) |
|  |  | Coeff. (SD) | Ref. | -373.1 (59.1) | -536 (64.9) | -627.3 (63.6) | -656.8 (57.7) |
|  |  | *p* value | Ref. | <0.001 | <0.001 | <0.001 | <0.001 |
|  | BIS_LT | Mean (SD) | 948.2 (238.6) | 602.2 (170.4) | 476.3 (136.4) | 400.1 (143.7) | 389.5 (160.8) |
|  |  | Coeff. (SD) | Ref. | -346.0 (61.4) | -471.9 (59.1) | -552.1 (64.7) | -579.8 (53.9) |
|  |  | *p* value | Ref. | <0.001 | <0.001 | <0.001 | <0.001 |
|  | BIS_LE | Mean (SD) | 976.8 (249.1) | 628.6 (196.7) | 494.3 (143.0) | 392.7 (146.2) | 360.5 (149.1) |
|  |  | Coeff. (SD) | Ref. | -348.3 (59.2) | -482.5 (62.9) | -590.9 (63.9) | -645.5 (55.7) |
|  |  | *p* value | Ref. | <0.001 | <0.001 | <0.001 | <0.001 |
|  | BrVis_F4 | Mean (SD) | 741.0 (388.3) | 455.5 (237.0) | 415.5 (278.6) | 244.5 (130.1) | 337.6 (293.9) |
|  |  | Coeff. (SD) | Ref. | -285.5 (106.3) | -325.5 (111.5) | -498.0 (84.5) | -419.2 (113.5) |
|  |  | *p* value | Ref. | 0.007 | 0.004 | <0.001 | <0.001 |
|  | BrVis_P4 | Mean (SD) | 670.4 (334.9) | 526.3 (204.8) | 437.4 (225.8) | 294.8 (161.8) | 290.6 (197.2) |
|  |  | Coeff. (SD) | Ref. | -144.1 (80) | -233 (72.6) | -378.9 (68.3) | -397.8 (77.2) |
|  |  | *p* value | Ref. | 0.072 | 0.001 | <0.001 | <0.001 |
|  | BrVis_F3 | Mean (SD) | 707.0 (446.9) | 450.0 (289.7) | 391.4 (213.1) | 224.2 (139.3) | 263.5 (289.8) |
|  |  | Coeff. (SD) | Ref. | -257(125.8) | -316 (10.2) | -481.9 (96.3) | -445.3 (101.4) |
|  |  | *p* value | Ref. | 0.041 | 0.009 | <0.001 | <0.001 |
|  | BrVis_P3 | Mean (SD) | 662.8 (307.5) | 527.8 (215.2) | 403.5 (188.5) | 298.7 (154.2) | 298.7 (192.0) |
|  |  | Coeff. (SD) | Ref. | -135.0 (66.4) | -259.4 (65.2) | -372.4 (64.9) | -376.7 (74.2) |
|  |  | *p* value | Ref. | 0.042 | <0.001 | <0.001 | <0.001 |

SD: Standard deviation

Supplementary Table 3. Descriptive data and relationship between haemodynamic and respiratory variables at baseline and in each of the recordings made in steep Trendelenburg.

| Parameter (Units) | | Time since steep Trendelenburg positioning | | | | |
| --- | --- | --- | --- | --- | --- | --- |
|  |  | Basal | 30min | 90min | 150min | 210min |
| HR | Mean (SD) | 60.8 (11.8) | 57.2 (7.9) | 60.0 (6.8) | 64.5 (8.3) | 64.7 (8.4) |
|  | *p* value | Ref. | 0.140 | 0.704 | 0.180 | 0.287 |
| SBP | Mean (SD) | 110.9 (19.3) | 123.2 (18.2) | 115.1 (15.9) | 120.9 (13.9) | 116.9 (15.6) |
|  | *p* | Ref. | 0.038 | 0.393 | 0.072 | 0.244 |
| EtCO2 | Mean (SD) | 33.5 (2.7) | 33.9 (1.9) | 34.7 (1.4) | 35.6 (2.0) | 35.4 (2.2) |
|  | *p* value | Ref. | 0.568 | 0.088 | 0.013 | 0.032 |
| MAC | Mean (SD) | 1.0 (0.1) | 1.0 (0.1) | 1.0 (0.1) | 1.0 (0.1) | 1.0 (0.1) |
|  | *p* value | Ref. | 0.769 | 0.218 | 0.146 | 0.342 |

HR: Heart Rate; SBP: Systolic Blood Pressure; EtCO2: End Tidal CO2; MAC: Minimum Alveolar Concentration; SD: Standard deviation.
