# Supplementary material for: Effects of sustained Trendelenburg position on the spectral signatures of the EEG: implications for the consistency of the level of anesthesia, an observational study
Source: J Clin Monit Comput. 2025 Dec 22;40(2):323–32. doi: 10.1007/s10877-025-01403-x (PMC13053590; doi:10.1007/s10877-025-01403-x)
Supplement: Supplementary file 4 — Supplementary Material 4 [file 10877_2025_1403_MOESM4_ESM.docx]

**APPENDIX 1: ANAESTHETIC MANAGEMENT OF ROBOTIC ASSISTED RADICAL PROSTATECTOMY**

MONITORING

- ECG, Non invasive Blood Pressure, SpO_2_, Capnography, Temperature
- Arterial line (invasive blood pressure)
- Train of four (TOF)
- BIS bilateral
- 5-Channel EEG (depending on Neurophysiology): F3-F4-C3-C4-CZ-Ref

INDUCTION

- NO Benzodiazepines
- Fentanyl IV: 1-1.5 mcg/kg
- Propofol IV: 1.5-2.5 mg/kg
- (or Etomidate IV: 0.2-0.4 mg/kg)
- Rocuronium IV: 0.6-1.2 mg/kg

MAINTENANCE

- Sevoflurane: MAC 0.8-1.2 (Target SEF 10-14 Hz)
- Rocuronium IV (Continuous infusion: 0.3-0.4 mg/kg/h
- (Optional) Remifentanil IV (Continuos infusion, 20/40 mcg/ml): TCI 0.5-4 mcg/ml
- (Optional) Fentanyl IV bolus: 50-100 mcg. Max. 0.5-0.7 mcg/kg
- (Optional) IV analgesia: Acetaminophen, metamizole, morphine

EDUCTION

- STOP Rocuronium continuous infusion after completion of urethral suture.
- Neuromuscular blockade verification (TOF):
- If TOF=4 & TOF>90%: No sugammadex needed.
- If TOF=3, or 4 & TOF <90%: Sugammadex 2 mg/kg.
- If TOF=2: Sugammadex 4 mg/kg.
- If TOF=1: Sugammadex 8 mg/kg.
- If TOF=0: Sugammadex 16 mg/kg.

*In obese patients, dosing is based on actual weight.

- Sugammadex administration and BIS-EEG recording after skin suture.
- Stop sevoflurane and remifentanil continuous infusion
- Analgesia.
- Extubation (optional).

BIS-EEG RECORDING

- BIS-EEG RECORDING PARAMETERS:
- MAC: 0.9-1 (adjusted to patient’s age) (Target SEF95: 10-14 Hz)
- TCI remifentanil: 2 mcg/ml
- BIS-EEG RECORDING MOMENTS:
  - Baseline after induction.
  - 30 minutes after steep Trendelemburg.
  - 90-150-210 minutes after steep Trendelemburg.
  - Just before reseting operating table back to initial position (0º)
  - 15-30 minutes after reseting operating table back to initial position (0º)
  - During sugammadex administration.

       *During data recording (2 minutes period recording EEG), try not to use electric scalpel.

**APPENDIX 2**

We used custom MatlabTM scripts developed by the research team were then used to import and process the files transferred from the BIS VISTATM for further analysis. The SEF95 and BIS index have a sampling rate of 1 Hz, while EEG data are sampled at 128 Hz. EEG signals through the BrainAmp amplifier (EEG-BrVis) signals were amplified and filtered from 0.3 to 1,000 Hz, sampled at 2,000 Hz with a resolution of 0.1 µV and a resolution of 0.1 µV, and stored for further analysis using MatlabTM.

For data analysis, 120 second blocks of the EEG-BIS signal and the EEG-BrVis signal in X, Y, Z were selected for analysis. Hereafter we estimated the BIS index and SEF95 means within the 2-minute blocks (i.e. 120 samples) for the left and right sides. For analysis of the EEG data (both EEG-BIS and EEG-BrVis), we followed the approach described by our previous investigations by Martínez-Simón et al.37,38 We estimated the spectral content of EEG signals between 0 and 45 Hz using a multitaper spectral analysis implemented with the mtspectrumc function of the Chronux applications using an 8-percent window. Chronux applications using an 8-second window overlapped at 75% with tapper parameters TW=4 and K=9, where TW is the time-bandwidth product and K is the number of tapes.

We decided to carry out the normalisation of the spectral values by dividing them by the spectrum mean (estimated over the whole set of spectra for each channel and subject, but for each channel and subject, but considering the X blocks) and multiplying by 100. Finally, the total power in each band [delta (1-4 Hz), theta (4-8 Hz), alpha (8-14 Hz), beta (14-35 Hz) and gamma (35-45 Hz)] was estimated by averaging the normalised values within the corresponding ranges.

To compute the area under the curve of the spectral power (PSD-AUC), the integral of the power spectral density was evaluated within the defined frequency range. The spectral slope (X) (power-law-slope) was estimated by fitting a linear function to the power spectrum represented in double-logarithmic units. In addition to the slope, the intercept of the fitted line with the power axis (b) (power-law-intercept) was also determined.
